# Respiratory physiotherapy for critically ill children: concern regarding a recommendation

**DOI:** 10.1186/s40560-024-00764-w

**Published:** 2024-12-18

**Authors:** Nobuaki Shime

**Affiliations:** Department of Emergency and Critical Care Medicine, Graduate School of Biomedical & Health Sciences, Hiroshima University, 1-2-3 Kasumi, Minami-ku, Hiroshima, 734-8551 Japan

## Abstract

The guideline entitled “Japanese Clinical Practice Guidelines for Rehabilitation in Critically Ill Patients 2023” was published by the Japanese Society of Intensive Care Medicine in 2023. However, there is an issue with the clinical question and recommendation for respiratory physiotherapy in mechanically ventilated children. Although the evidence was based on two randomized controlled trials regarding prone positioning, the recommendation may have risk of misunderstanding as a recommendation for all respiratory physiotherapy. There are abundant evidence-based recommendations against chest physiotherapy for infants with bronchiolitis with no benefit and possible adverse events. Revising the recommendation for respiratory physiotherapy in critically ill, mechanically ventilated children should be considered.

**To the Editor:**

I read with considerable interest the guideline entitled “Japanese Clinical Practice Guidelines for Rehabilitation in Critically Ill Patients 2023” published by the Japanese Society of Intensive Care Medicine in 2023 (J-ReCIP 2023) [1]. This is a first-ever, excellent guideline advocating the importance of rehabilitation for critically ill patients. This guideline provides standardized recommendations based on a rigorous systematic review using the GRADE (Grading of Recommendations, Assessment, Development, and Evaluation) methodology, guiding physicians, nurses and physiotherapists toward better practice in the ICU setting.

However, among the proposed recommendations, the specific recommendation for respiratory physiotherapy in critically ill children is a concern. As shown in the graphical abstract in Fig. 2 of the guideline, the term of respiratory physiotherapy may lead to recall chest physiotherapy such as percussion, vibration, quick compression of the thorax or abdomen during expiration to increase expiratory flow. Unfortunately, however, the authors performed a meta-analysis of only two randomized, controlled trials of prone positioning intervention compared with supine positioning as those met the PICO criteria. This results in inconsistency between the title of the clinical question and the content of evidence.

A Cochrane review of chest physiotherapy in children with bronchiolitis [2] including 12 randomized controlled trials concluded that chest physiotherapy does not decrease the severity or time to recovery, the duration of oxygen supplementation, or the length of stay. More importantly, forced expiratory physiotherapy techniques were associated with adverse events, such as respiratory destabilization and vomiting. Although the Cochrane review [2] included neonates and infants from 0 to 24 months who required hospitalization owing to bronchiolitis, which might not be identical to the “Patient” of PICO in the J-ReCIP 2023 guideline, the abovementioned adverse effects may play a greater effect on critically ill children. Several other guidelines strongly recommend against routinely implementing chest physiotherapy in infants with acute bronchiolitis [3, 4].

Consequently, I suggest rephrasing the title of clinical question 12 (and even the symbol in Fig. 2 as indicating hand-assisted physiotherapy) to better represent the contents of existing evidence regarding the effects of prone positioning intervention compared with supine positioning. This suggestion could lead to avoiding misunderstanding by users who tend to follow the recommendation while not referring to the rationale in depth and to saving vulnerable children from unnecessary and dangerous physical interventions being applied.

## Data Availability

Data sharing is not applicable to this article because no datasets were generated or analyzed during the current study.

## References

[1] Unoki T, Hayashida K, Kawai Y, Taito S, Ando M, Iida Y, et al. Japanese clinical practice guidelines for rehabilitation in critically ill patients 2023 (J-ReCIP 2023). J Intensive Care. 2023;11(1):47. 37932849 10.1186/s40560-023-00697-w PMC10629099

[2] Roqué i Figuls M, Giné-Garriga M, Granados Rugeles C, Perrotta C, Vilaró J. Chest physiotherapy for acute bronchiolitis in paediatric patients between 0 and 24 months old. Cochrane Database Syst Rev. 2016; 2: CD004873. 10.1002/14651858.CD004873.pub5 PMC6458017 26833493

[3] National Institute for Health and Clinical Excellence. Bronchiolitis in children: diagnosis and management. London: The Institute; 2015. https://www.nice.org.uk/guidance/ng9. Accessed 10 Nov 2024.

[4] O’Brien S, Borland ML, Cotterell E, Armstrong D, Babl F, Bauert P, et al. Paediatric research in emergency departments international collaborative (PREDICT) network, Australasia. Australasian bronchiolitis guideline. J Paediatr Child Health. 2019;55(1):42–53. 30009459 10.1111/jpc.14104

